# Gravidität bei vorbestehendem Diabetes (Update 2023)

**DOI:** 10.1007/s00508-023-02188-2

**Published:** 2023-04-20

**Authors:** Alexandra Kautzky-Willer, Yvonne Winhofer, Raimund Weitgasser, Monika Lechleitner, Jürgen Harreiter

**Affiliations:** 1 Gender Medicine Unit, Abt. für Endokrinologie und Stoffwechsel, Universitätsklinik für Innere Medizin III, Medizinische Universität Wien, Währinger Gürtel 18–20, 1090 Wien, Österreich; 2 Abteilung für Innere Medizin/Diabetologie, Privatklinik Wehrle-Diakonissen, Salzburg, Österreich; 3 Universitätsklinik für Innere Medizin I, LKH Salzburg – Universitätsklinikum der Paracelsus Medizinischen Privatuniversität, Salzburg, Österreich; 4 Avomed-Arbeitskreis für Vorsorgemedizin und Gesundheitsförderung in Tirol, Innsbruck, Österreich

**Keywords:** Präkonzeptionell bestehender Diabetes, Typ 1 Diabetes mellitus, Typ 2 Diabetes mellitus, Adipositas, Schwangerschaft, Schwangerschaftsplanung, Diabetische Embryopathie, Diabetische Komplikationen, Perinatale Morbidität, Pre-gestational diabetes, Type 1 diabetes mellitus, Type 2 diabetes mellitus, Obesity, Pregnancy, Pre-pregnancy care, Diabetic embryopathy, Diabetic complications, Perinatal morbidity

## Abstract

Bereits vor mehr als 30 Jahren forderte die St. Vincent Deklaration, dass Schwangere mit vorbestehendem Diabetes mellitus vergleichbare Schwangerschaftsergebnisse wie gesunde Frauen erreichen sollen. Dennoch bestehen bei Frauen mit konzeptionell manifestem Diabetes nach wie vor höhere Komplikationsraten und eine höhere perinatale Morbidität und Mortalität. Eine fehlende oder zumindest unzureichende Schwangerschaftsplanung und präkonzeptionelle Betreuung mit Optimierung der Stoffwechsellage vor Konzeption ist dafür verantwortlich. Alle Frauen mit Diabetes sollen im Selbstmanagement der Insulintherapie mit Anpassungen der Insulindosis geschult sein und eine stabile Stoffwechsellage vor Schwangerschaftsbeginn aufweisen. Eine Schilddrüsendysfunktion, Hypertonie oder diabetische Komplikationen vor der Konzeption sollten ausgeschlossen bzw. adäquat behandelt sein, um das Risiko einer möglichen Progression der Komplikationen und Begleiterkrankungen sowie insgesamt mütterliche und fetale Risiken zu minimieren. Ein Ziel der mütterlichen Stoffwechselkontrolle ist das Erreichen von Normoglykämie und normalen HbA_1c_-Werten, falls dies ohne Risiko für Hypoglykämien möglich ist, da eine schlechte Blutzuckereinstellung mit diabetischer Embryopathie und diabetischer Fetopathie assoziiert ist. Das Hypoglykämierisiko ist speziell bei Typ 1 Diabetes mellitus in der Frühschwangerschaft deutlich erhöht, nimmt aber mit den hormonellen Veränderungen und der Zunahme der Insulinresistenz im Schwangerschaftsverlauf deutlich ab. Eine weltweit steigende Adipositasprävalenz führt zusätzlich zu einem Anstieg von Müttern mit Typ 2 Diabetes. Dieser Trend ist auch bei Frauen mit Typ 1 Diabetes zu beobachten und aggraviert die Metabolik und die perinatalen Ergebnisse. Eine funktionelle, intensivierte Insulintherapie mit multiplen täglichen Insulininjektionen oder eine Insulinpumpentherapie tragen neben dem vermehrten Einsatz des kontinuierlichen Glukosemonitorings zum Erreichen einer guten mütterlichen Stoffwechselkontrolle vor und während der Schwangerschaft bei. Orale Antidiabetika (Metformin) können vor allem bei Typ 2 Diabetes und Adipositas helfen die Insulinsensitivität zu verbessern und dadurch den Insulinbedarf zu vermindern, sollten jedoch aufgrund der Plazentagängigkeit und ungewissen Langzeitergebnissen bei den Nachkommen mit Bedacht (shared decision making) verordnet werden. Aufgrund des erhöhten Präeklampsierisikos bei Frauen mit Diabetes in der Schwangerschaft ist hier ein frühes Screening zu empfehlen. Regelmäßige und engmaschige geburtshilfliche Kontrollen in einem spezialisierten Zentrum und bei spezialisierten Fachärzt:innen sowie interdisziplinäre Zusammenarbeit werden empfohlen um eine gute Stoffwechseleinstellung und gesunde Entwicklung des Kindes zu sichern.

## Abstract (en)

In 1989 the St. Vincent Declaration aimed to achieve comparable pregnancy outcomes in women with diabetes and those with normal glucose tolerance. However, currently women with pre-gestational diabetes still feature a higher risk of perinatal morbidity and even increased mortality. This fact is mostly ascribed to a persistently low rate of pregnancy planning and pre-pregnancy care with optimization of metabolic control prior to conception. All women should be experienced in the management of their therapy and on stable glycemic control prior to conception. In addition, thyroid dysfunction, hypertension as well as the presence of diabetic complications should be excluded or treated adequately before pregnancy in order to decrease the risk for a progression of complications during pregnancy as well as maternal and fetal morbidity. Near normoglycaemia and HbA_1c_ in the normal range are targets for treatment, preferably without the induction of frequent resp. severe hypoglycaemic reactions. Especially in women with type 1 diabetes mellitus the risk of hypoglycemia is high in early pregnancy, but it decreases with the progression of pregnancy due to hormonal changes causing an increase of insulin resistance. In addition, obesity increases worldwide and contributes to higher numbers of women at childbearing age with type 2 diabetes mellitus and adverse pregnancy outcomes. Intensified insulin therapy with multiple daily insulin injections and pump treatment are equally effective in reaching good metabolic control during pregnancy. Insulin is the primary treatment option. Continuous glucose monitoring often adds to achieve targets. Oral glucose lowering drugs (Metformin) may be considered in obese women with type 2 diabetes mellitus to increase insulin sensitivity but need to be prescribed cautiously due to crossing the placenta and lack of long-time follow up data of the offspring (shared decision making). Due to increased risk for preeclampsia in women with diabetes screening needs to be performed. Regular obstetric care as well as an interdisciplinary treatment approach are necessary to improve metabolic control and ensure the healthy development of the offspring.

## Grundsatz-Statement

In der St. Vincent Deklaration wurde 1989 als Ziel festgelegt, dass Frauen mit Diabetes in Zukunft vergleichbare Schwangerschaftsergebnisse haben sollen wie stoffwechselgesunde Frauen. Dennoch weisen Frauen mit Diabetes nach wie vor mehr mütterliche und kindliche Komplikationen und eine höhere perinatale Mortalität auf. Dies liegt vor allem an der immer noch unzureichenden Schwangerschaftsvorbereitung und Blutzuckeroptimierung zu Beginn der Gravidität. Auch während der Gravidität einschließlich der Geburt muss eine möglichst normoglykämische Stoffwechsellage gewährleistet sein. Nach Möglichkeit sollten Frauen mit Diabetes von einem in der Behandlung schwangerer Frauen mit Diabetes erfahrenen, interdisziplinären Team an einem spezialisierten Zentrum betreut werden. Die Entbindung sollte an einer Abteilung mit neonataler Intensivstation erfolgen.

## Schwangerschaftsplanung – perikonzeptionelle Betreuung

Eine Schwangerschaft bei Frauen mit manifestem Diabetes betrifft immer noch hauptsächlich Frauen mit Typ 1 Diabetes mellitus (T1DM). Rezente Erhebungen zeigen aber auch eine kontinuierliche Zunahme des Typ 2 Diabetes mellitus (T2DM), der zusätzlich zur Hyperglykämie durch die adipositas-bedingten Risiken und oft auch durch ein höheres mütterliches Alter kompliziert wird [1–3]. Selbst bei den Schwangeren mit T1DM wird im letzten Jahrzehnt ein signifikanter Anstieg des BMI festgestellt [3]. Sowohl bei T1DM als auch T2DM waren neben der Stoffwechselkontrolle und Diabetesdauer zu Schwangerschaftsbeginn ein höherer mütterlicher BMI und ein höherer Blutdruck mit schlechteren Schwangerschaftsergebnissen verbunden [4]. Frauen mit Migrationshintergrund sowie Frauen aus niedrigen sozialen Schichten machen einen beträchtlichen Anteil der Frauen mit T2DM aus, insbesondere bei jener Gruppe, die vor der Gravidität unzureichend behandelt und auf die Schwangerschaft vorbereitet war oder bei der ein vorbestehender Diabetes überhaupt erst in der Schwangerschaft neu entdeckt wurde. Persistierend schlechte Schwangerschafts-Outcomes bei Frauen mit präkonzeptionellem Diabetes werden auch in aktuellen Populations-basierten Erhebungen bestätigt [5]. Mütterliche Adipositas und unzureichende Stoffwechseleinstellung sind die wesentlichen veränderbaren (mütterliches Alter, Diabetesdauer und Deprivation die wichtigsten nicht modifizierbaren) Risikofaktoren. Keine Unterschiede fanden sich bei kongenitalen Fehlbildungen und Totgeburten zwischen Frauen mit Typ 1 und Typ 2 Diabetes, während Frühgeburten und LGA Neugeborene bei Typ 1 Diabetes häufiger auftraten [5]. Frauen mit Typ 2 Diabetes wiesen allerdings eine höhere neonatale Mortalitätsrate auf. Ein HbA_1c_ ≥ 6,5 % (48 mmol/mol) im 3. Trimester, Typ 2 Diabetes und eine soziale Schlechterstellung der Mütter waren unabhängige Risikofaktoren für den perinatalen Tod. Verstärkte Beachtung ist auf die optimale Glykämie der Frauen mit Typ 2 Diabetes vor und in der Schwangerschaft zu legen, da diese Patientinnen oft bereits zusätzliche kardiovaskuläre Risikofaktoren, Komorbiditäten und medikamentöse Therapie aufweisen und das Komplikationsrisiko oft unterschätzt wird [6].

**Figure 1:**
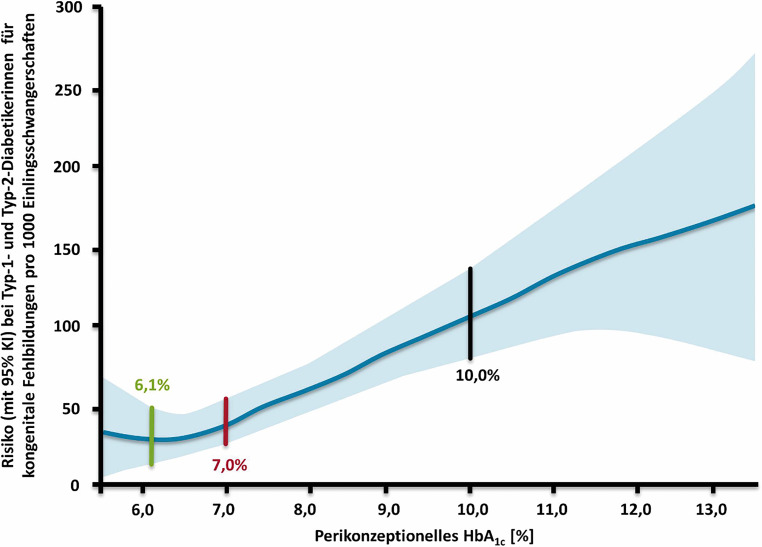

Frauen mit Diabetes müssen – unabhängig von der Diabetesform – eine Schwangerschaft planen, um optimale Voraussetzungen für die kindliche Entwicklung, aber auch die eigene Gesundheit zu gewährleisten und das Risiko für perinatale Komplikationen zu reduzieren [1, 2]. Frauen mit Diabetes sollten bezüglich der Verhütungsmethode dieselben Optionen wie gesunden Frauen zur Verfügung stehen, da das Risiko einer ungeplanten Schwangerschaft das Risiko der Kontrazeption übertrifft [7]. In einer großen amerikanischen Studie mit Frauen mit T1DM und T2DM konnte die Sicherheit von hormonellen Verhütungsmethoden und niedriges Risiko für thromboembolische Ereignisse (1 thromboembolischer Event pro 100 Patientinnen-Jahre) gezeigt werden, wobei das niedrigste Risiko bei Verwendung von intrauterinen und implantierbaren subdermalen kontrazeptiven Methoden beobachtet wurde [8]. Das Hauptproblem bei vorbestehendem Diabetes ist die Entstehung einer diabetischen Embryopathie [1, 2]. Aus diesem Grund werden eine prinzipielle präkonzeptionelle Beratung und engmaschige Betreuung aller Frauen mit vorbestehendem Diabetes und Kinderwunsch oder Schwangerschaft empfohlen. Um Missbildungen und Aborte zu vermeiden soll der Glukosestoffwechsel mit einem HbA_1c_ < 6,5 %, zumindest aber unter 7 % bereits bei Kinderwunsch optimiert sein [7, 9]. Generell sollte versucht werden Normoglykämie zu erreichen, sofern dies ohne Hypoglykämien möglich ist. Bei einem HbA_1c_ über 8 % ist das Risiko für Morbidität und Mortalität deutlich erhöht (Tab. 1, Abb. 1). Die perikonzeptionelle Stoffwechselkontrolle und eine Nephropathie waren in einer populationsbasierten Kohortenstudie die wichtigsten unabhängigen Prädiktoren für das Auftreten von kindlichen Anomalien, die insgesamt 3‑ bis 6‑fach häufiger waren als bei gesunden Schwangeren [10]. Dabei stieg das Fehlbildungsrisiko linear an (nahezu 30 % per 1 % HbA_1c_-Anstieg über 6,3 %). Das Hypoglykämierisiko ist insbesonders im 1. Trimenon sehr hoch und muss individuell berücksichtigt werden, sinkt dann aber mit zunehmender Insulinresistenz deutlich. In einem systematischen Review wurde pro 1 % HbA_1c_-Absenkung eine Reduktion des relativen Risikos für kongenitale Fehlbildungen um 0,39–0,59 bei Frauen mit T1DM oder T2DM ermittelt [11]. Um eine normoglykämische Stoffwechsellage zu erreichen, sollen die Frauen in funktioneller Insulintherapie geschult oder in der Insulinpumpentherapie erfahren sein. Prinzipiell sind die beiden Therapieformen in ihrer Effektivität vergleichbar. Dies wird in einem Cochrane Review bei allgemein schlechter Studienqualität bestätigt [12]. Aufgrund des Fortschritts in der Pumpentechnologie wird die Durchführung von neuen, qualitativ hochwertigen Studien empfohlen. Derzeit ist von den in Österreich erhältlichen closed loop Systeme nur ein System in der Schwangerschaft zugelassen. Eine multizentrische Studie mit fast 400 Schwangerschaften mit Frauen mit Typ 1 Diabetes zeigte unter einer Basis-Bolus-Therapie im Vergleich zu einer Pumpentherapie niedrigere HbA_1c_-Werte für letztere bei vergleichbarem Hypoglykämierisiko [13]. Eine rezente Analyse der CONCEPTT Studie zeigte allerdings ein schlechteres Outcome von schwangeren Frauen mit Insulinpumpentherapie im Vergleich zu Basis-Bolus Therapie [14]. Unter einer intensivierten Basis Bolus Therapie wurden bessere glykämische Parameter, sowie ein geringeres Risiko für Schwangerschaftshypertonie, neonatale Hypoglykämie und Aufnahmen auf die neonatale Intensivstation festgestellt.

**Table 1:** 

HbA_1c_ [%] ^a^ (mmol/mol)	Kongenitale Fehlbildungen [%]	RR (95 % KI) ^b^	Perinatale Mortalität [%]	RR (95 % KI) ^b^
< 6,9 (< 52)	3,9	1,4 (0,8–2,4)	2,1	2,8 (1,3–6,1) ^c^
6,9–7,8 (52–62)	4,9	1,8 (1,0–2,9)	2,8	3,8 (1,9–7,3) ^c^
7,9–8,8 (63–73)	5,0	1,8 (0,9–3,3)	3,3	4,4 (2,0–9,4) ^c^
8,9–10,3 (74–89)	3,9	1,4 (0,6–3,1)	6,3	8,3 (4,2–15,9) ^c^
≥ 10,4 (≥ 90)	10,9	3,9 (1,8–7,8) ^c^	5,5	7,3 (2,5–19,8) ^c^
Durchschnittsbevölkerung	2,8	1,0	0,75	1,0

*RR* relatives Risiko

^a^ Standard Referenzwert 5,4 ± 1,0 % (36 ± 11 mmol/mol) (Mittelwert ± 2 SD) in der gesunden Durchschnittsbevölkerung

^b^ im Vergleich zur Durchschnittsbevölkerung

^c^ Signifikant höher als in der Durchschnittsbevölkerung (Signifikanzniveau 0,05)

Komplexere Therapiekonzepte sollten bereits frühzeitig präkonzeptionell bei Kinderwunsch geschult und erlernt werden, um eine Gravidität bei stabiler normoglykämischer Stoffwechsellage zu ermöglichen. Der Einsatz der Pumpentherapie in Kombination mit Continuous Glucose Monitoring System (CGMS) Messungen kann die Einstellung in der Schwangerschaft erleichtern. In der CONCEPTT Studie konnte bei der Verwendung von CGMS in der Schwangerschaft oder der Zeit der Schwangerschaftsplanung bei Frauen mit T1DM gezeigt werden, dass ein besseres neonatales Outcome im Vergleich zur kapillären Blutzuckerselbstmessung mit signifikant weniger LGA Geburten, Hypoglykämien und Aufenthalten auf der neonatologischen Intensivstation und kürzerem Spitalsaufenthalt verbunden ist [15]. Ebenso konnte eine signifikant geringere Zeit mit hyperglykämischen Blutzuckerwerten und dabei mehr Zeit im Zielbereich, bei nur minimalen Änderungen des HbA_1c_ und vergleichbaren Hypoglykämieraten beobachtet werden. Die sichere und verlässliche Anwendung eines Flash Glucose Systems im Vergleich zu Blutzuckerselbstmessung wurde in der Schwangerschaft bei T1DM, T2DM und Gestationsdiabetes beobachtet [16]. Wichtig ist die korrekte Einstellung des Zielbereichs bei kontinuierlicher Blutzuckermessung. Dieser liegt zwischen 63–140 mg/dl bei schwangeren Frauen und sollte > 70 % (Time in Range) im Zielbereich bei schwangeren Frauen mit T1DM und wenn möglich noch höher bei T2DM und GDM sein [17]. Die Zeit unter dem Zielbereich (Time below Range) sollte so gering wie möglich sein, aber zumindest < 4 %.

Bei bestehendem Kinderwunsch ist eine Abklärung diabetischer Spätkomplikationen dringend erforderlich (Tab. 2). Eine Kontrolle des Augenhintergrundes beim Augenfacharzt, Kontrolle der Nierenfunktion und falls erforderlich weitere Abklärung durch einen spezialisierten Facharzt, die Einstellung des Blutdrucks mit in der Schwangerschaft geeigneten Medikamenten (potenziell teratogene Eigenschaften von Medikamenten wie ACE Hemmer, AT1-Rezeptor-Blocker, Statine, usw. beachten), eine Abklärung koronarer Herzkrankheit, Dyslipidämie, ein Ausschluss einer Schilddrüsenfunktionsstörung, sowie eine Gewichtsreduktion bei Adipositas sollten bei Kinderwunsch bestenfalls vor dem Absetzen von Verhütungsmitteln erfolgen [3, 18]. Im ersten Trimenon sollte ein TSH Wert unter 2,5 μU/L angestrebt werden, in jedem Fall aber ist bei schwangeren Frauen mit positiven TPO Antikörpern oder TSH Werten über 10 µU/L ein sofortiger Therapiebeginn mit Schilddrüsen-Hormonen empfohlen [19]. Die Einnahme von Folsäure (mindestens 400 μg/Tag) bereits bei Kinderwunsch bis einschließlich der 12. Schwangerschaftswoche ist obligat. Bei Adipositas oder T2DM werden bis zur 12. Schwangerschaftswoche sogar höhere Dosen (5 mg) empfohlen [20].

**Table 2:** 

Insulintherapie	Funktionelle Insulintherapie (Basal-Bolus-Prinzip) oder Insulinpumpe werden präferiert
	Der Wechsel auf komplexere Dosierungsformen sollte möglichst vor Beendigung von Verhütungsmethoden erfolgen
Hypoglykämierisiko	Kann limitierend für eine optimale Therapieeinstellung sein
	Besonders in der Frühschwangerschaft bei T1DM ist das Risiko besonders hoch (3–5 × erhöht) [22]
Folsäure	Beginn mit Folsäurepräparat drei Monate vor Beenden der Verhütung [18]
Augenkontrollen	Kontrolle beim Spezialisten bei Kinderwunsch (Fundus)
	Bei Retinopathie ist falls erforderlich eine Therapieeinleitung durchzuführen. Der Kinderwunsch sollte bis zur Stabilisierung einer Retinopathie und Erreichen der Glukoseziele verzögert werden
	Kontrolle: wenn möglich präkonzenptionell/bei Kinderwunsch, jedes Trimester, 3 Monate postpartum, danach individuell je nach Erfordernis (zumindest 1 × /Jahr)
Nierenfunktion	Stadieneinteilung nach Nephropathieklassifikation
	Bei Niereninsuffizienz Risikoab- und -aufklärung durch spezialisierte/*n* /Fachärztin/Facharzt vor Absetzen der Verhütungsmethoden. Regelmäßige Kontrollen (1 × /Trimeneon) in der Schwangerschaft – Screening auf Albuminurie
Neurologische Komplikationen	Möglich bei langer Diabetesdauer und schlechter Blutzuckereinstellung
	Evtl. Vorliegen von Hypoglykämiewahrnehmungsstörungen, diabetischer Gastroparese oder orthostatischer Hypotonie
Makrovaskuläre Komplikationen	Schwangerschaft steigert kardiovaskuläres Risiko bei Frauen mit Diabetes
	Erhöhtes Risiko bei: langer Diabetesdauer, höherem Alter, Nikotinkonsum, art. Hypertonie, fam. Hyperlipidämie, positiver Familienanamnese bzw. bei bereits stattgehabten kardiovaskulärem Ereignis, diabetischer Nephropathie
Blutdruck	Zielwert: 110–140/75–85 mm Hg, Kontraindikation: ACE Hemmer + AT1-Rezeptor-Blocker
	Abklärung einer KHK, wenn vorhanden: Risikoab- und -aufklärung und gegebenenfalls Therapieeinleitung
	Erhöhtes Präeklampsierisiko: Acetylsalicylsäure (Aspirin®) (100–150 mg/Tag) zur Prävention, Beginn bei erhöhtem Risiko vor der 16. Schwangerschaftswoche bis zur 36. Schwangerschaftswoche empfohlen
Lipide	Statine, Bempedoinsäure, PCSK9-Hemmer, Fibrate und Niacin kontraindiziert
	Gallesäurebindende Substanzen prinzipiell möglich, aber Nebenwirkungen (Gastrointestinaltrakt) beachten. Schwache Evidenz, in Kasuistiken wurde außerdem ein erhöhtes Risiko von fetalen intrakraniellen Blutungen aufgrund eines Vitamin K Mangels beschrieben [23]
Endokrine Abklärung	Messung von TSH und TPO Antikörper vor Schwangerschaft
	Bei Übergewicht: Gewichtsreduktion vor Schwangerschaft empfohlen (5–10 %)

## Gravidität

Während der Schwangerschaft soll versucht werden individualisiert die bestmögliche Stoffwechsellage unter Berücksichtigung der Hypoglykämie-Wahrnehmung und -häufigkeit, der individuellen Fähigkeiten so wie der Lebensumstände mit normoglykämischen Blutzuckerwerten (Tab. 3) zu erreichen. Lebensstilmaßnahmen mit regelmäßiger körperlicher Aktivität und Ernährungsumstellung sind auch in der Schwangerschaft zu empfehlen und eine diätologische Beratung soll den schwangeren Frauen mit Diabetes angeboten werden (siehe Kapitel Gestationsdiabetes). Generell ist bei Frauen mit präkonzeptionellem Diabetes mellitus eine Insulintherapie in der Schwangerschaft zu empfehlen [7]. Gerade zu Beginn der Schwangerschaft ist die Hypoglykämierate relativ groß und die Insulindosis vorsichtig anzupassen. Insbesonders bei Frauen mit Typ 1 Diabetes ist das Risiko für schwere Hypoglykämien in der Frühschwangerschaft 3‑ bis 5‑fach höher als vor der Schwangerschaft [22]. In der CONCEPTT Studie zeigten 30 % der Schwangeren mit Typ 1 Diabetes eine verminderte Hypoglykämiewahrnehmung, was mit mehr hypoglykämischen Episoden, größerer Glukosevariabilität und Hypoglykämie-Angst/Diabetes Distress trotz CGM verbunden war[24]. Generell gilt, dass im Lauf der Gravidität (üblicherweise beginnend mit der 20. Schwangerschaftswoche) die Tagesdosis auf 50–100 %, bei adipösen Frauen mit Typ 2 Diabetes oft noch höher angehoben werden müssen, um die zunehmende Insulinresistenz zu kompensieren und die empfohlenen Blutzuckerzielwerte in der Schwangerschaft zu erreichen (Tab. 3). Frauen mit T2DM und Kinderwunsch sollten bereits präkonzeptionell auf eine Insulintherapie umgestellt werden. Im Falle einer ungeplanten Gravidität bei T2DM unter Einnahme oraler Antidiabetika (OAD) gibt es bisher keine Evidenz für ein erhöhtes Missbildungsrisiko durch OAD, jedoch ist zu bedenken, dass Metformin plazentagängig ist und es bezüglich Langzeitfolgen bei den Nachkommen noch wenige Erkenntnisse vorliegen [7, 25]. Bei sehr insulinresistenten und stark übergewichtigen Frauen mit T2DM kann aber eine zusätzliche Therapie mit Metformin überlegt werden, um den Stoffwechsel zu verbessern und die Insulinresistenz zu mildern [26]. Bei langzeitiger Einnahme von Metformin und Schwangerschaft wird eine Vitamin B12 Kontrolle empfohlen [7, 25]. Für den Einsatz anderer OADs kann keine Empfehlung abgegeben werden. Eine Umstellung von OAD auf Insulin und eine entsprechende Schulung der Patientinnen zur Selbstanpassung der Insulindosis und Aufklärung über mögliche Risiken, sowie die zu erwartenden Stoffwechselveränderungen in der Schwangerschaft sollten durch die betreuenden Ärztinnen und Ärzte bei Schwangerschaftsplanung/Kinderwunsch erfolgen. Bevorzugt werden die kurzwirksamen Insulinanaloga Insulin Aspart und Insulin Lispro verabreicht und mittlerweile in der Regel gegenüber Normalinsulin präferiert eingesetzt. Studien zeigen die sichere Anwendbarkeit von Insulin Lispro und Insulin Aspart in der Schwangerschaft [27, 28]. Die ultraschnell-wirksamen Insuline Aspart (Fiasp®) und Lispro (Lyumjev®) sind in der Schwangerschaft ebenso zugelassen. Für Glulisin liegen in der Gravidität derzeit nur Vigilanzdaten vor [29], die keine besonderen Auffälligkeiten zeigen. Aufgrund der schlechten Datenlage wird eine Anwendung in der Schwangerschaft nicht empfohlen.

**Table 3:** 

Zeitpunkt der Blutzucker-Selbstmessung	Blutzucker (mg/dl)
Nüchtern und vor den Mahlzeiten	65–95
1 h nach Beginn der Mahlzeit	< 140
2 h nach Beginn der Mahlzeit	< 120
Vor dem Schlafen gehen, ca. 22:00–23:00 Uhr	90–120
Nachts in der Zeit von 2:00–4:00 Uhr	> 60
Zeit im Zielbereich 63–140 mg/dl	> 70 %

Einige langwirksame Insulinanaloga sind in der Schwangerschaft zugelassen (Insulin Glargin, Insulin Glargin U300, Insulin Detemir) und können sicher angewendet werden, jedoch zeigten bisherige Daten keine eindeutigen Vorteile von Insulin Glargin oder Insulin Detemir gegenüber einer Therapie mit NPH-Insulinen bei T1DM oder T2DM [30]. In einer Vergleichsstudie zwischen Detemir und NPH-Insulinen wurden vergleichbare HbA_1c_-Werte und ein ähnliches Hypoglykämierisiko beschrieben [30, 31]. Die Detemirgruppe konnte jedoch signifikant niedrigere Nüchternwerte in der 24. und 36. Schwangerschaftswoche vorweisen [30]. Auch in der Analyse der perinatalen Komplikationen gab es zwischen den Insulin Glargin und Insulin Detemir vergleichbare Resultate [32]. Eine rezente Metaanalyse von Insulin Glargin Daten mit etwa 700 diabetischen schwangeren Frauen verglichen zu NPH zeigt vergleichbare maternale und kindliche Ergebnisse [33]. Eine Behandlung mit Insulin Degludec kann – falls medizinisch indiziert – während der Schwangerschaft in Betracht gezogen werden. Studien zu Insulin Degludec in der Schwangerschaft beschreiben eine sichere Anwendbarkeit und vergleichbare Outcomes verglichen zu bereits länger etablierten Basalinsulinen [34–36].

Während der Geburt sind Blutzuckerwerte zwischen 90–126 mg/dl anzustreben [37]. Nach Entbindung ist eine rasche Reduktion der Insulindosen um etwa 50 % und enge Blutzuckerkontrolle erforderlich, da die Insulinsensitivität rasch zunimmt [38].

Diabetische Folgeerkrankungen wie eine Retinopathie, Nephropathie oder autonome Neuropathie können fortschreiten, wobei die Veränderungen meist postpartal reversibel sind und im Langzeitverlauf somit üblicherweise durch die Gravidität selbst keine Progression eintritt. Eine diabetische Retinopathie kann erstmalig in der Schwangerschaft auftreten, aber auch eine Progression in der Schwangerschaft ist möglich [21]. Es handelt sich um die häufigste mikrovaskuläre Komplikation bei Diabetes in der Schwangerschaft, weswegen regelmäßige Kontrollen empfohlen werden (Tab. 2). Risikofaktoren für eine Progression sind schlechte perikonzeptionelle Blutzuckerkontrolle, lange Diabetesdauer (> 10 Jahre), diabetische Nephropathie, arterielle Hypertonie, unzureichende Vorbehandlung einer diabetischen Retinopathie beziehungsweise eine ungünstige Ausgangslage, Nikotinkonsum und die hormonellen Veränderungen in der Schwangerschaft an sich, aber auch eine Anämie [21].

Frauen mit Nephropathie haben ein deutlich erhöhtes Risiko für die Entwicklung einer Präeklampsie, Frühgeburt sowie eine Wachstumsretardierung des Kindes. Im Falle bereits vor der Schwangerschaft bestehender Spätkomplikationen muss eine Aufklärung der schwangeren Frau über ihr Risiko erfolgen. Während der Gravidität und postpartal sollte eine engmaschige, regelmäßige Beobachtung der Patientin durchgeführt werden (Tab. 2). Eine Schwangerschaft an sich führt nicht zur Abnahme der Nierenfunktion. Falsch positive Befunde können beispielsweise bei schlechter Stoffwechselkontrolle oder Harnwegsinfekt auftreten [21]. Erhöhtes maternales und fetales Risiko besteht bei erhöhtem Serumkreatinin > 1,5 mg/dl, einer Nephropathie Stadium 3 sowie unkontrollierter oder schwer behandelbarem Bluthochdruck. Eine interdisziplinäre Betreuung in Kooperation mit spezialisierten Nephrolog:innen ist zur Risikoreduktion essenziell.

An eine diabetische Neuropathie soll vor allem bei Frauen und Kinderwunsch mit langjähriger Diabetesdauer gedacht werden.

Diabetes in der Schwangerschaft ist mit einem erhöhten Präeklampsie-Risiko verbunden. Daher sollte eine präventive Anwendung von niedrig dosiertem Aspirin (100–150 mg/Tag) zwischen der 12. und 16. Schwangerschaftswoche begonnen werden, um die Morbidität, Mortalität und auch Kosten zu senken [7, 39]. In den Leitlinien der Deutschen Diabetesgesellschaft wird eine Beendigung der Therapie bis zur 36. SSW basierend auf einer rezenten randomisiert kontrollierten Studie zur Verringerung von Blutungsrisiken empfohlen [37, 40]. Bei schwangeren Frauen mit Diabetes und Hypertension sind Blutdruckzielwerte zwischen 110–140/75–85 mm Hg anzustreben [7, 41]. Als hypertensive Werte gelten Blutdruckwerte > 140/90 mm Hg [41].

## Interessenkonflikt

A. Kautzky-Willer hat von folgenden Unternehmen, die auch fördernde Mitglieder der ÖDG sind, Forschungsunterstützungen/Honorare erhalten: Eli Lilly, Novo Nordisk. Y. Winhofer hat von folgenden Unternehmen, die auch fördernde Mitglieder der ÖDG sind, Forschungsunterstützungen/Honorare erhalten: Sanofi Aventis, Eli Lilly, Astra Zeneca, Novartis. R. Weitgasser hat von folgenden Unternehmen, die teils auch fördernde Mitglieder der ÖDG sind, Forschungsunterstützungen und/oder Honorare erhalten: Abbott Diabetes Care, Amgen, Astra Zeneca, Boehringer-Ingelheim, Daiichi Sankyo, Eli Lilly, MSD, Novo Nordisk, Roche Diabetes Care, Sanofi. M. Lechleitner hat von folgenden Unternehmen, die auch fördernde Mitglieder der ÖDG sind, Honorare erhalten: Astra Zeneca, Bristol-Myers Squibb, Germania Pharmazeutika, GlaxoSmithKline Pharma, Eli Lilly, Medtronic, Merck Sharp & Dome, Novartis, Novo Nordisk, Pfizer, Sanofi-Aventis, Takeda. J. Harreiter gibt an, dass kein Interessenkonflikt besteht.
